# Correlations between gustatory, trigeminal, and olfactory functions and nasal airflow

**DOI:** 10.1007/s00405-023-07962-6

**Published:** 2023-05-02

**Authors:** Anna Kristina Hernandez, Antje Walke, Antje Haehner, Mandy Cuevas, Thomas Hummel

**Affiliations:** 1 Department of Otorhinolaryngology, Smell and Taste Clinic, TU Dresden, Haus 5, Fetscherstrasse 74, 01307 Dresden, Germany; 2 Department of Otolaryngology – Head and Neck Surgery, Philippine General Hospital, University of the Philippines - Manila, Manila, Philippines; 3 Department of Otolaryngology – Head and Neck Surgery, Asian Hospital and Medical Center, Muntinlupa, Philippines

**Keywords:** Smell, Taste, Trigeminal, Olfactory, Chemosensory tests, Nasal airflow

## Abstract

**Purpose:**

To determine the relationship of chemosensory screening and nasal airflow tests among the same set of participants, and to determine other factors that are related to the outcomes of these tests.

**Methods:**

Participants had no chemosensory complaints. Structured medical history was taken. Participants underwent 5 screening tests: q-sticks (orthonasal olfaction), q-powders (retronasal olfaction), trigeminal lateralization test, taste sprays, and peak nasal inspiratory flow (PNIF). Ratings of smell/taste ability and nasal airflow were obtained using visual analogue scales (VAS). Composite sinusitis symptoms and significance of olfaction questionnaire scores were also determined.

**Results:**

Four hundred participants were included in the study, 156 men, 244 women; aged 18–82 years (mean: 46). The q-powders and taste spray scores were weakly positively correlated with all the other chemosensory tests and PNIF. However, chemosensory test scores were not correlated with VAS, composite sinusitis symptoms, and significance of olfaction questionnaire scores. Various tests showed significant decrease starting at specific ages (in years, PNIF and trigeminal lateralization: 40, q-powders: 60, and q-sticks: 70).

**Conclusion:**

Chemosensory screening tests and self-rated chemosensory function showed no correlation in participants without chemosensory complaints. In addition, gustatory function appeared to be correlated with olfactory and trigeminal function but also with nasal airflow, and nasal airflow was related not only to olfactory but also to trigeminal and taste function. Over all, the results suggest that chemosensory functions (orthonasal olfactory, trigeminal, retronasal olfactory, gustatory) and nasal airflow are correlated with each other, which we propose may be possibly mediated, at least in part, through central nervous system interactions.

## Introduction

In recent years, there has been increasing interest on the chemical senses, particularly as they can be impaired in those with a history of COVID-19 infection. However, studies have focused more on the relationship between the various types of chemosensory dysfunctions [1–3]. Less is known about the relationship of the chemical senses with each other, especially in the absence of any chemosensory complaint [4, 5].

Olfaction, gustation, trigeminal function, and nasal airflow are all anatomically bound to the oral-nasal region and are functionally interrelated. The flow of air through the nose facilitates odorants to reach the area of the olfactory mucosa. Orthonasal and retronasal olfaction depend on airflow, with the former being anteroposterior in direction and primarily for sensation of smells in the environment, while the latter being posteroanterior and primarily for sensation of vapors from the back of the mouth when eating or drinking [6–8]. In this way, retronasal olfaction and trigeminal inputs (temperature, texture, pungency) influence the perception of flavor and are associated with the sense of taste [6, 9, 10]. At the same time, most odors also elicit both olfactory and trigeminal sensations, especially when presented at higher concentrations [6, 11–14]. The trigeminal nerve also signals sensations of pain, temperature and touch in the nose and mouth, while also influencing olfaction and perception of nasal patency [10, 15–17].

This study aimed to determine the relationship of olfaction, gustation, trigeminal function, and nasal airflow with each other in individuals without chemosensory complaints. Due to the large sample we aimed to test, we decided to use peak nasal inspiratory flow (PNIF, nasal airflow) and various chemosensory screening tests (q-sticks (orthonasal olfaction) [18], q-powders (retronasal olfaction) [19], trigeminal lateralization (trigeminal function) [20], and taste sprays (whole mouth gustation) [21]) to measure function. In addition, the study aimed to determine whether self-ratings (measured using visual analogue scales, VAS) for smell ability, taste ability, and nasal airflow, composite sinusitis symptom scores, significance of olfaction questionnaire scores, or other patient-related factors are related to the outcomes of these screening tests.

## Materials and methods

The cross-sectional study design was approved by the Institutional Review Board at the University Clinic of the TU Dresden (application number BO-EK-201052020) and was conducted according to the principles expressed in the Declaration of Helsinki. Possible risks and benefits related to participation in the study were explained to participants during the initial consultation. All participants provided their written informed consent.

### Participants

The study included individuals of at least 18 years of age without any chemosensory complaints who presented for testing at a private dental clinic. A standardized structured history was taken [22] including the following: age, sex, height, weight, history of smoking/alcohol consumption/chemical exposure/head injury/headaches, rhinologic symptoms (episodes of frequent sinusitis, allergic rhinitis, postnasal drip, frequent cold, nasal obstruction, runny nose, nasal polyps, and snoring), and presence of co-morbid conditions (nerve/brain disease, diabetes mellitus, hyper/hypothyroidism, hepatitis, kidney disease). VAS ratings, composite sinusitis symptoms, and significance of olfaction questionnaire scores were also determined. Participants with incomplete data were not included in selected analyses.

Five tests were investigated in this study, namely:

### Screening Tests

#### Q-sticks (3-item orthonasal odor identification test)

In the q-sticks test [23], three odors (cloves, coffee, and rose) are presented in felt-tip pens similar to those used in the “Sniffin’ Sticks” test [24]. These 3 odors were selected because they are widely known and their identification is not strongly dependent upon subjects’ age [18]. The highest score is 3.

#### Trigeminal lateralization test

This test was conducted based on how it was done in a study by Frasnelli et al. [20], using 2 squeezable bottles pressed simultaneously to deliver an airstream into both nostrils, but only for a total of 10 times. Only one of the bottles contains 20 ml Eucalyptol (order number C80601; Sigma Aldrich, Taufkirchen, Germany) and participants must identify the which side of the nostril was presented with this substance. The highest score is 10.

#### Peak nasal inspiratory flow (PNIF)

PNIF is a measure of nasal airflow and was measured using the peak flow meter (Inspiratory flow meter, order number 3109750; Clement Clarke Int. Ltd., Harlow, UK). The test was done twice, with each participant asked to inhale deeply through both nostrils each time. The higher value of the two attempts was recorded.

#### Q-powders (3-item retronasal olfaction test)

The q-powders test [19] comprised three odors (cinnamon, banana, garlic; Givaudan Schweiz AG, Dubendorf, Switzerland). Participants were asked to choose which among the 6 descriptors, presented as flash cards, best describes the flavor of each of the powders. The odors were selected based on results from previous studies where the identification rates of the 3 selected odors were high (> 95%) [25]. The highest score is 3, a score of 0 may be interpreted as anosmia, while 1 or 2 would mean that further testing is required [19].

#### Taste spray total score (4 item whole mouth taste test)

Similar to Vennemann et al. [21], four basic tastes (sweet, sour, salty, and bitter) were tested using approximately 0.1 ml/spray, 1–2 sprays on the middle of the tongue. Participants were asked to identify the taste according to a list of 4 taste descriptors. After each sample, participants rinsed their mouth with water. Based on clinical experience, impaired taste function was assumed if the score was less than 3 [26].

Other measures of function:

### Other Measures of Function

#### Visual analogue scale (VAS) rating for smelling ability, tasting ability, and nasal airflow

Participants were asked to rate their smelling ability, tasting ability, and nasal airflow from 0 to 10, with a score of 10 being the highest.

#### Composite sinusitis symptom score

This score is the combined score of the following (1 point each): Frequent sinusitis, allergic rhinitis, postnasal drip, frequent colds, nasal polyps, nasal obstruction, runny nose, and snoring; with a maximum score of 8.

#### Significance of olfaction questionnaire

Based on the work by Croy et al. [27], a modified 20-item questionnaire, in German, including items related to association, application, and consequence of sense of smell was administered to participants. A sum of the scores for each subtest (each item ranged from a score of 1–4) and the total score of all items and subtests were used in the analysis.

### Data collection and statistical analysis

Patient records were assigned codes and anonymized. Data were encoded into a Microsoft Excel Office 365 version 2107 database (Microsoft Corp., Redmond, WA, USA) and checked for accuracy of encoding. Data analysis was done using SPSS software (Version 28.0; IBM Corp., Armonk, NY, USA). Pearson’s r, spearman’s rho, and t-tests were used in the analysis of the data, with a p value of < 0.05 considered significant.

## Results

**Fig. 1:**
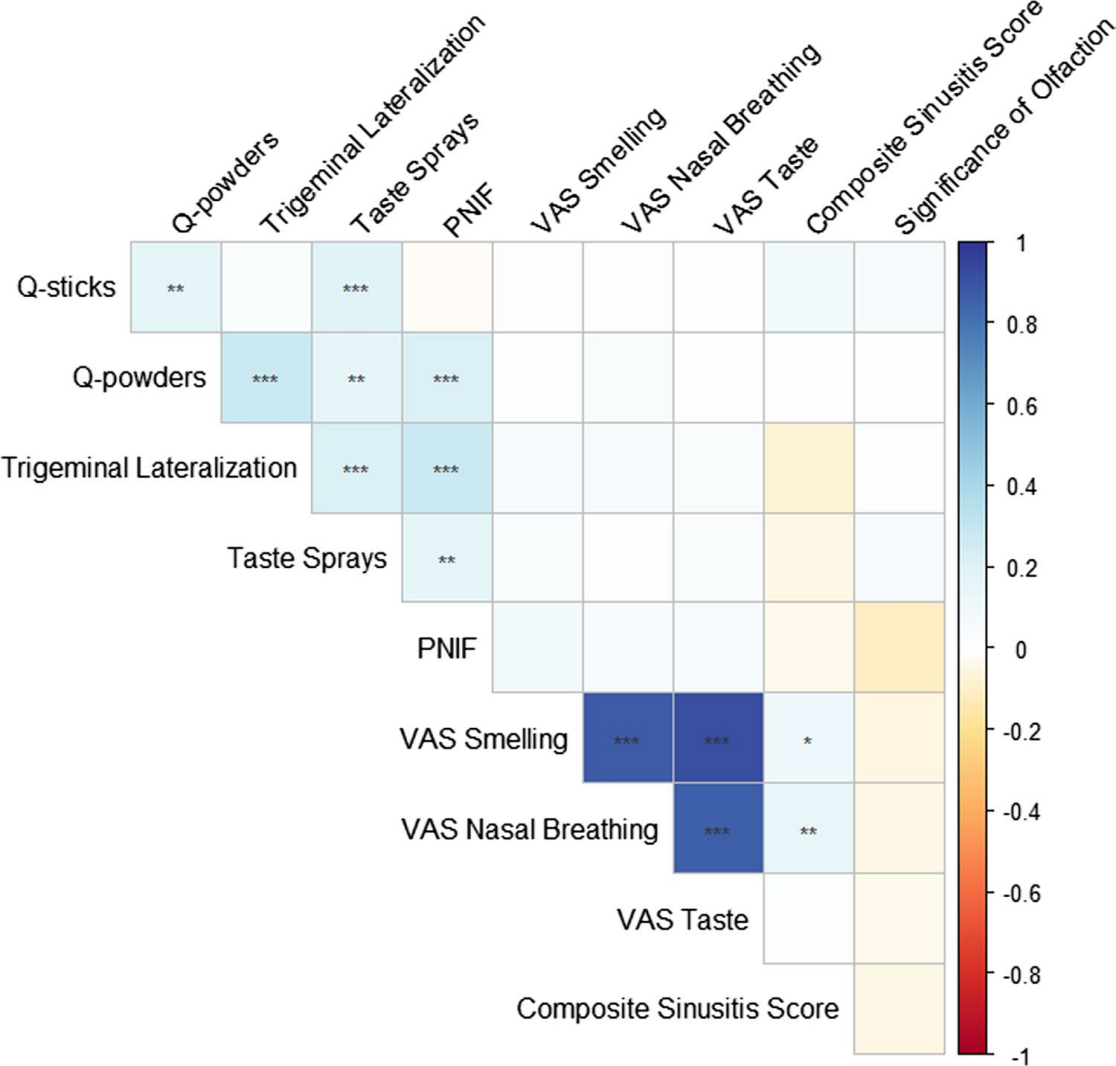
Correlation between various chemosensory tests, PNIF, visual analogue scale (VAS) ratings, composite sinusitis symptom scores, and significance of olfaction questionnaire scores. Legend: box colors denote strength of correlation (blue: positive correlation, red: negative correlation), * p < 0.05, ** p < 0.01, ***p < 0.001

Results are summarized in Tables 1 and Fig. 1. Four hundred participants were included in the study, 156 men, 244 women, aged 18–82 years (mean: 46 years). Men (mean = 141.8, n = 153) had higher PNIF compared to women (mean = 118.5, n = 242, t_253.082_ = 4.37, p < 0.001). There were no significant differences between the genders for the chemosensory screening tests.

**Table 1:** Differences of chemosensory tests among various age levels

Age range	Maximum n^a^	q-sticks	Trigeminal lateralization	PNIF	q-powders	Taste sprays
≥ 18	395					
≥ 30	347					
≥ 40	250		X	X		
≥ 50	164		X	X		
≥ 60	91		X	X	X	
≥ 70	19	X	X	X	X	

*X* Those with this age and older have significantly lower scores

^a^Maximum n: maximum number of participants analyzed for a particular age group

**Fig. 2:**
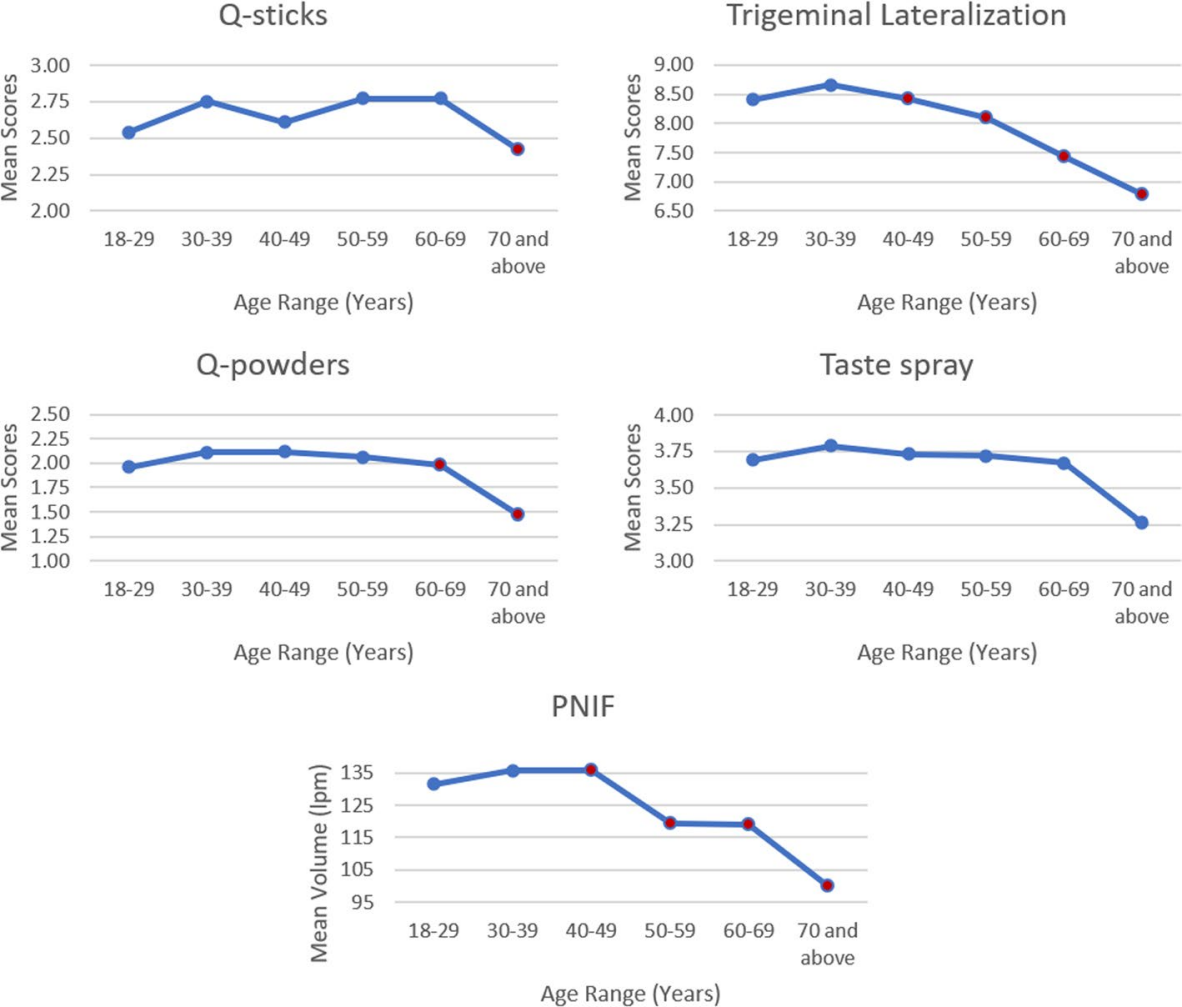
Mean values for chemosensory screening and nasal airflow tests among different age groups. Significantly lower scores in this age group and older

Age was negatively correlated with trigeminal lateralization (r_392_ = – 0.21, p < 0.001) and PNIF (r_395_ = – 0.18, p < 0.001), but not with the other tests. Differences of chemosensory test scores across the ages are summarized in Table 1 and in Fig. 2. Those aged 40 and older had lower trigeminal lateralization (t_390_ = 2.58, p = 0.01) and lower PNIF (40 and older: t_393_ = 2.14, p = 0.033). Those aged 60 and older had lower q-powders scores (t_391_ = 2.03, p = 0.044), while those aged 70 and older also had lower q-sticks scores (t_391_ = 2.11, p = 0.035).

Height (r_395_ = 0.21, p < 0.001) and weight (r_393_ = 0.12, p = 0.022) were also correlated with PNIF but not with any of the chemosensory screening tests.

Of the factors gathered from participants’ personal history, we noted several significant findings. However, due to the low number of participants belonging to some groups, we decided to report results only from groups with n ≥ 20. Q-powders scores were higher for those with exposure to chemicals (n = 69, t_364_ = 3.03, p = 0.003), while trigeminal lateralization (n = 80, t_106.088_ = 2.80, p = 0.006) and taste spray (n = 78, t_93.685_ = 2.06, p = 0.043) scores were lower for those with history of snoring. History of smoking, alcohol consumption, head injury, allergic rhinitis, postnasal drip, frequent colds, nasal obstruction, runny nose, and other co-morbid conditions such as diabetes mellitus, hyperthyroidism, and hypothyroidism had no effect on chemosensory screening tests or PNIF. Variables other than those that have been mentioned had significant results but the group sizes were less than 20 for each.

The q-powders and taste spray scores were weakly positively correlated with all the other chemosensory tests and PNIF (Fig. 1). The Q-sticks and trigeminal lateralization tests were weakly positively correlated with q-powders and taste spray total scores, and with PNIF for the latter. PNIF was weakly positively correlated with trigeminal lateralization, q-powders, and taste spray total scores. Chemosensory test scores were not correlated with self-ratings (VAS), but a significantly higher taste spray total score was observed in individuals with VAS taste ability ratings ≥ 9 (n = 13, t376 = 9.38, p < 0.001), VAS smell ability ratings ≥ 8 (n = 58, t101.138 = 2.06, p = 0.042), and VAS nasal airflow ratings ≥ 6 (t231.770 = 2.05, p = 0.042).

Composite sinusitis symptom scores and significance of olfaction questionnaire scores (including subtests and total scores) were not correlated with chemosensory screening tests or PNIF (Fig. 1). There was a tendency, though, for those with a score of ≥ 1 on the composite sinusitis symptom score to have lower q-sticks scores (t_359.043_ = 1.937, p = 0.53). When exploring for possible relationship of significance of olfaction questionnaire scores to chemosensory test scores, only those with higher significance of olfaction (90th percentile, score of ≥ 71, n = 41) had a significantly higher q-sticks score (t_60.136_ = 2.49, p = 0.016).

## Discussion

Several studies have investigated the relationship between the different chemical senses (orthonasal/retronasal olfaction, trigeminal function, gustation) and there have been some conflicting findings.

A study by Fonteyn et al. found that orthonasal and retronasal function are correlated among those with post-infectious, post-traumatic, idiopathic, toxic, and neurologic conditions [30], while Hummel et al. found higher intensities for retronasal versus orthonasal stimulus presentation in healthy individuals [31]. It has also been proposed that differing airflow patterns between orthonasal and retronasal flow may result in lower concentration of odors reaching the olfactory cleft [32]. Furthermore, it has also been proposed that orthonasal and retronasal stimuli are processed differently, given that odors may be presented at higher concentrations retronasally due to greater intraoral odor release from salivation, warming, and mastication [33, 34]. On the other hand, several studies have also documented individuals with poor orthonasal function in the presence of normal retronasal function [8, 29]. Conversely, evidence also exists in support of synergistic relationship between various chemical senses. For instance, orthonasal and trigeminal co-stimulation have been found to improve trigeminal localization [35]. Blankenship et al. also found that retronasal, but not orthonasal odors, share processing circuitry commonly associated with taste; and that orally-sourced (retronasal) olfactory input is processed by a brain region responsible for taste processing, whereas externally-sourced (orthonasal) olfactory input is not [36].

Interestingly, age was negatively correlated to trigeminal lateralization and PNIF, but not to the other tests. This may support the presence of a progressive age-related decline in intranasal trigeminal sensitivity and lung function [40–42]. Perhaps the few items present in orthonasal, retronasal, and whole mouth taste screening tests also precluded having enough variation in scores to determine a trend. Worth noting, however, was that the different chemical senses appear to begin deteriorating at specific ages. Trigeminal function and nasal airflow both appear to be the first to diminish, followed by retronasal, orthonasal, and lastly – gustation.

Our study has shown the presence of a weakly positive correlation of q-sticks (orthonasal test) and trigeminal lateralization (trigeminal function) to both q-powders (retronasal test) and taste spray total scores (whole mouth taste test), as well as PNIF (nasal airflow) for the latter, which seems to be in support of a relationship between these senses. This is partly consistent with a study by Migneault-Bouchard et al. [1] where they noted correlations between scores for olfaction, gustation, and trigeminal function. They found that olfactory loss leads to a decrease in taste and trigeminal sensation (compare with [2, 14]), instead of a compensation through hyperfunction of other chemical senses [1]. Another study by Han et al. found a similar interaction among the chemical senses, where patients with olfactory dysfunction showed increased electric taste thresholds and decreased scores for the umami taste strip [3].

In contrast, however, we did not find a correlation between q-sticks and trigeminal lateralization in our study. Although a complex interaction, both synergistic and antagonistic, has been found in previous studies between trigeminal and olfactory function [11, 37], screening tests and a cohort of only healthy individuals may not be the most ideal in confirming this relationship due to inherent limitations previously mentioned and further discussed later.

Retronasal and whole mouth taste tests were both weakly positively correlated to all other chemosensory tests and to PNIF. This reinforces the relationship of the various chemical senses, even airflow, in the appreciation of taste and flavor. The sensation of flavor is known to be a combined experience involving the sense of taste that is enhanced by retronasal olfaction. But it is also interesting that trigeminal sensation through somatosensation (temperature, texture, etc.) may also contribute in the appreciation of both taste and flavor [38] and our findings seem to be in support of this interaction (see also [39]).

Although we found significant findings depicting interactions between history of chemical exposure and q-powders, as well as between snoring, trigeminal lateralization, and taste spray scores, some of these findings contradict what has previously been published in literature. For instance, chronic chemical exposure has been found to have adverse effects on human olfaction and is supported by findings in animal experiments [43]. However, we found that those with history of chemical exposure performed better in the q-sticks test in our study. We are unsure if previous chemical exposure leads to heightened retronasal sensation or if both the orthonasal and retronasal screening tests failed to discriminate well between varying levels of function when administered to healthy individuals, leading to these unusual findings. Snoring is one of the prominent symptoms of obstructive sleep apnea (OSA), with OSA having an incidence of 20–70% among snoring patients [44]. Previous studies also showed that snoring was associated with adverse effects on peripheral nerve function [45, 46]. However, a study by Heiser et al. [46] found no significant difference between taste strip scores and nasal trigeminal lateralization scores of those with and without OSA. Despite having more testing repetitions for trigeminal lateralization (40 compared to our 10), their sample size was smaller (n = 44). On the other hand, snoring may also be due to an altered balance of nasal and oral airflow from chronic rhinosinusitis (CRS) [47], as snoring also has increased prevalence among those with CRS [48]. Given both OSA and CRS are diseases associated with increased inflammation, this chronic state of inflammation, that can also be present in subclinical CRS, may result in increased production of inflammatory cytokines that may affect both the sense of smell and taste. Inflammation has been proposed to be toxic to olfactory neurons, causing potentially irreversible changes to the mucosa and resulting in the disturbance of olfactory mucus that may affect odor transduction [49–51]. Furthermore, inflammation has also been proposed to trigger apoptosis and abnormal cell turnover in taste buds, possibly leading to problems with taste transduction and ultimately causing taste dysfunction [52]. We are uncertain if our findings reflect true relationships or are simply an overestimation of the presence of relationships due to limitations of screening tests as a method for evaluation in healthy individuals. It appears to be prudent to reassess these factors using more comprehensive psychophysical tests and regard the present findings as a pilot in this direction which needs further confirmation despite the large sample size.

The relationship of sex and height to PNIF may be secondary to men and taller individuals being more likely to have larger lung capacity [53–55], leading to greater nasal inspiratory flow. The finding that nasal airflow is correlated with trigeminal lateralization is somewhat expected, given that trigeminal sensation serves as a means to protect the airway from potentially harmful substances which can then lead to shortening or cessation of inspiration reflexively [14, 15]. But the correlation of PNIF to q-powders and taste spray total scores may emphasize the role of nasal airflow in the perception of taste and flavor. Unexpectedly, there was no correlation between q-sticks and PNIF. The relationship between nasal airflow obstruction and olfaction has been frequently studied in literature [56–61], particularly in patients with sinunasal disease. However, our sample was comprised of healthy individuals and it is possible that this relationship between olfaction and airflow was not clearly depicted in this population. Also, although a relationship between olfaction and airflow through the olfactory cleft has been mentioned previously in literature [62, 63], PNIF measures airflow through the entire nasal cavity and not only to the olfactory cleft on maximal inspiration, and may also be confounded by the influence of lung function. For these reasons, we may not have observed a correlation between the two tests. We propose that these may be better explored using comprehensive orthonasal olfactory tests involving both healthy participants and those with olfactory loss.

Self-ratings of chemosensory function have been shown to be unreliable, at least in portions of the patients [28]. In the clinical setting, patients tend to classify an olfactory impairment with accompanying retronasal olfactory issues as a taste dysfunction [9, 29]. In the present study among healthy individuals, we found that VAS scores were also not correlated with any of the chemosensory screening tests or with PNIF. Although those with higher VAS ratings for smell / taste ability and nasal airflow had significantly higher taste spray scores, we attribute this finding to the limitation of screening tests to discriminate between varying degrees of function or dysfunction, given that the number of items are very few. Although self-ratings may be helpful in determining symptom burden in those with chemosensory dysfunction, the value of self-ratings in estimating olfactory function in healthy people is limited. This emphasizes the value of psychophysical testing, especially preceding any nasal surgical intervention, for a more accurate estimation of olfactory function.

We hypothesized that decreased chemosensory function may not simply be due to actual decrease in function but perceived importance of the lost function to an individual, such that similar deficits may be reported as varying in severity depending on value placed on the senses. However, there was no correlation between significance of olfaction subtest and total scores to any of the chemosensory screening tests. It was interesting, though, that those who had higher significance of olfaction scores also had higher q-sticks scores, which could also confirm that people who value their sense of smell also tend to perform better on psychophysical tests, particularly those that require attention and cognitive ability [24, 64].

Chemical senses are rarely experienced in isolation and various studies have shown activation of similar brain regions when it comes to taste and oral somatosensory stimuli (anterior insula), as well as olfaction, oral texture of food, and perception of umami (orbitofrontal cortex) among others [38, 65, 66]. It remains uncertain how or where the integration of various chemical senses occurs exactly, but we propose that central nervous system processing may play a role in the integration of inputs from the different chemical senses.

### Limitations

Smell and taste screening tests were created to facilitate more efficient assessment of olfactory and gustatory function, and these have been quite useful in clinical practice. However, there remain to be challenges when psychophysical chemosensory tests are shortened for ease and efficiency of testing. Shorter tests may not distinguish between varying degrees of function and dysfunction [67] and may have limited or overestimated some findings in our analyses. For example, the q-sticks test only has 3 items and there is no established distinction between what it means when scores vary from 0 to 3. It may be useful in screening for olfactory loss, especially if an individual scores 0 in the test. However, there is a possibility of false alarms, where individuals of normal olfactory function score less than 3. In addition, as much as one-third of those with a perfect score of 3 in the q-sticks test may still have abnormal orthonasal olfactory function [23]. Also, having fewer items may also influence how much chance performance affects outcomes [67]. Future studies may replicate our methodology but comparing more comprehensive orthonasal, retronasal, and taste psychophysical tests with trigeminal lateralization and nasal airflow measurements in patients both with and without olfactory loss.

## Conclusion

Chemosensory screening tests and self-rated chemosensory function showed little or no correlation in participants without chemosensory complaints. In addition, gustatory function appeared to be correlated with olfactory and trigeminal function, and nasal airflow was related not only to olfactory but also to trigeminal function. Overall, the results suggest that chemosensory functions (orthonasal olfactory, trigeminal, retronasal olfactory, gustatory) and nasal airflow are correlated with each other, which we propose may be possibly mediated, at least in part, through central nervous system interactions.

## Data Availability

The data that support the findings of this study are available from the corresponding author upon reasonable request.
